# Inclusion of private sector in district health systems; case study of private drug shops implementing modified Integrated Community Case Management (iCCM) strategy in Rural Uganda

**DOI:** 10.1186/1472-6963-14-S2-P65

**Published:** 2014-07-07

**Authors:** Freddy Kitutu, Chrispus Mayora, Phyllis Awor, Forsberg Birger, Stefan Peterson, Henry Wamani

**Affiliations:** 1 Makerere University College Health Sciences, Kampala, Uganda; 2 Karolinska Institute, Stockholm, Sweden

## Background

Uganda Ministry of Health passed the Public Private Partnership for Health (PPPH) policy to strengthen the health system by leveraging strategic advantages of private healthcare providers [1]. The National Malaria program has gone further to develop a malaria case management strategy through a multi-stakeholder consultative process [2]. Makerere University School of Public Health (MakSPH) partnered with Mbarara district to implement the iCCM strategy in private licensed drug shops in rural areas. The partnership aimed to increase access to quality medicines and point of care diagnostics for child febrile illnesses, minimize excess use of antimalarials and antibiotics, share information of cases diagnosed and treated at the drug shops and promote child survival.

## Methods

This was a plausibility design study with baseline and end-line assessments in the intervention and comparison districts. The intervention was introducing modified iCCM strategy at licensed drug shops in the rural Mbarara district. This involved training the drug shop attendants on how to manage febrile illnesses in under-fives using the standardised sick child job aid, supply of subsidized medicines and diagnostics, integration of drug shop health information system with district HMIS and routine support supervision. Qualitative interviews to explore views, attitudes and perceptions of various stakeholders and wider health systems effects of intervention are ongoing. Ethical approval was sought and granted.

## Results

Baseline surveys show that drug shops provide care to over 50% of child febrile illnesses in rural Uganda. 96 drug shop attendants were trained and hence 69 drug shops in rural counties of Mbarara district are implementing the modified iCCM strategy. Continuous monitoring and support supervision has started to explore how private drug shops can be integrated into the district health system. Drug seller performance and attrition, linkages with nearest public health facility and monthly reporting on pre-determined HMIS indicators are being examined.

## Conclusion

Private drug shops provide healthcare to under-five febrile children as first point of contact in rural areas. Their recognition and integration into district health systems will increase penetration of life saving interventions especially for the most vulnerable populations.
